# Reversal of experimental colitis disease activity in mice following administration of an adenoviral IL-10 vector

**DOI:** 10.1186/1476-9255-2-13

**Published:** 2005-10-31

**Authors:** Makoto Sasaki, J Michael Mathis, Merilyn H Jennings, Paul Jordan, Yuping Wang, Tomoaki Ando, Takashi Joh, J Steven Alexander

**Affiliations:** 1 Department of Molecular and Cellular Physiology, LSU Health Sciences Center, Shreveport, LA, 71130-3932, USA; 2 Department of Cell Biology and Anatomy, LSU Health Sciences Center, Shreveport, LA, 71130-3932, USA; 3 Department of Gastroenterology, LSU Health Sciences Center, Shreveport, LA, 71130-39322, USA; 4 Department of Obstetrics and Gynecology, LSU Health Sciences Center, Shreveport, LA, 71130-39322, USA; 5 Department of Internal Medicine and Bioregulation, Nagoya City University Graduate School of Medical Sciences, Nagoya, Japan

## Abstract

Genetic deficiency in the expression of interleukin-10 (IL-10) is associated with the onset and progression of experimental inflammatory bowel disease (IBD). The clinical significance of IL-10 expression is supported by studies showing that immune-augmentation of IL-10 prevents inflammation and mucosal damage in animal models of colitis and in human colitis. Interleukin-10 (IL-10), an endogenous anti-inflammatory and immunomodulating cytokine, has been shown to prevent some inflammation and injury in animal and clinical studies, but the efficacy of IL-10 treatment remains unsatisfactory. We found that intra-peritoneal administration of adenoviral IL-10 to mice significantly reversed colitis induced by administration of 3% DSS (dextran sulfate), a common model of colitis. Adenoviral IL-10 (Ad-IL10) transfected mice developed high levels of IL-10 (394 +/- 136 pg/ml) within the peritoneal cavity where the adenovirus was expressed. Importantly, when given on day 4 (after the induction of colitis w/DSS), Ad-IL10 significantly reduced disease activity and weight loss and completely prevented histopathologic injury to the colon at day 10. Mechanistically, compared to Ad-null and DSS treated mice, Ad-IL10 and DSS-treated mice were able to suppress the expression of MAdCAM-1, an endothelial adhesion molecule associated with IBD. Our results suggest that Ad-IL10 (adenoviral IL-10) gene therapy of the intestine or peritoneum may be useful in the clinical treatment of IBD, since we demonstrated that this vector can reverse the course of an existing gut inflammation and markers of inflammation.

## I. Introduction

Endothelial cell adhesion molecules ('*ECAMs*') play essential roles in the development of chronic inflammation by recruiting leukocytes, especially lymphocytes, to tissues. ECAMs support several forms of leukocyte adhesion including rolling, firm adhesion and extravasation [1]. Infiltration of tissues by leukocytes is a common hallmark of many chronic inflammatory states that include the inflammatory bowel diseases (IBD), ulcerative colitis (UC), and Crohn's disease (CD). In the setting of IBD, the expression of ECAMs like ICAM-1, VCAM-1, and MAdCAM-1 is observed in experimental models of colitis, and also within the inflamed human colon in Crohn's disease and ulcerative colitis [2-6].

Among the adhesion molecules up-regulated in IBD, MAdCAM-1, the mucosal cell adhesion molecule, is thought to be preeminent in the development of chronic gut inflammation. MAdCAM-1 is normally expressed in the gut, and its expression is dramatically amplified during inflammation [2,3]. The functional significance of increased appearance of MAdCAM-1 in IBD is supported by several reports which show that immunoneutralization of either MAdCAM-1 or its ligand, the α4β7 integrin, attenuate inflammation and mucosal damage in animal models of colitis [7-9]. However, since monoclonal antibodies directed against other ECAMs, particularly VCAM-1, can as well reduce disease activity in colitis models, the literature suggests that MAdCAM-1 is probably necessary, but insufficient for the maximal penetrance of experimental and probably also clinical IBD [10-13].

Based on these findings, it is apparent that a better understanding of the mechanisms regulating ECAM expression, especially that of MAdCAM-1, might help to devise improved therapies for colitis.

It has been suggested that pathologic activation of the mucosal immune system in response to antigens is a key factor in the pathogenesis of IBD. Furthermore, changes in leukocyte migration and cytokine production appear to contribute to the perpetuation of IBD [14]. Based on modern advances, recombinant anti-inflammatory cytokines (i.e. IL-10) treatment is now being developed for experimental colitis and human IBD. IL-10 produced by macrophages and monocytes appears to limit chronic inflammation [15-17], through several mechanisms including inhibition of the release of several inflammatory factors (IL-1, IL-6, IL-12, TNF-α, GM-CSF, GCSF), suppression of cell adhesive determinants (MHC class II molecule, β7), and by blocking ICAM-1 induction [18-24]. Conversely, IL-10 gene-knockout mice develop a chronic colitis that is extremely similar to IBD [25]. IL-10 treatment can reduce inflammation in several models of colitis and human IBD [26,18-34]. However, the clinical efficacy of systemically administered IL-10 for patients with mild to moderately active Crohn's disease has not been as effective as hoped [31-34]. Furthermore the efficacy of IL-10 administration in mouse colitis models is variable and model-specific [35].

We have previously described that exogenous IL-10 in vitro can block the expression of MAdCAM-1 in response to TNF-α, and attenuates lymphocyte adhesion to lymphatic node derived endothelium under cytokine stimulating conditions via NF-kB inhibition [36]. The purpose of the current study was to show that induction of endothelial expression of IL-10 through an IL-10 expression vector attenuates MAdCAM-1 expression in response to TNF-α and optimistically suggests the possibility of targeted Th2-cytokine gene therapy in IBD.

## II. Results

### A. Measurement of human IL-10 concentration in lavage fluids from the transfected peritoneum

**Figure 1:**
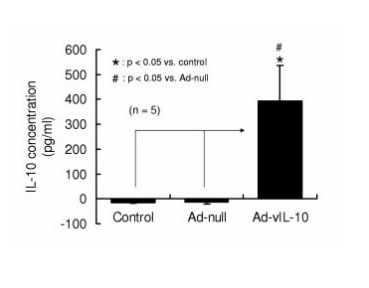
**IL-10 concentration in lavage fluids from the transfected peritoneum**. ELISA measurement of IL-10 in peritoneal lavage fluids from control shows a very high level of expression of IL-10 at approximately 400 pg/ml. No IL-10 was detected in lavage fluids of control or adeno-null mice (n = 5).

To screen for the efficacy of adenovirus mediated production of IL-10 in transfected mice, we measured the IL-10 concentration in the lavaged peritoneum in untreated, in adeno-'null' treated mice and in adeno-IL-10 transfected mice. There was no detectable human IL-10 signal in the non-transfected lavage fluid (control), nor was any mouse IL-10 detected (*data not shown*). However, the lavage fluid from the adenoviral IL-10 transfected mice showed a large and signficant increase in the IL-10 concentration (395 ± 136 pg/ml at 48 h after IL-10 gene transfection (Figure 1). Importantly, IL-10 was not detected in serum samples from these mice.

### B. Reduced disease activity in adeno IL-10 gene transfected mice

**Figure 2:**
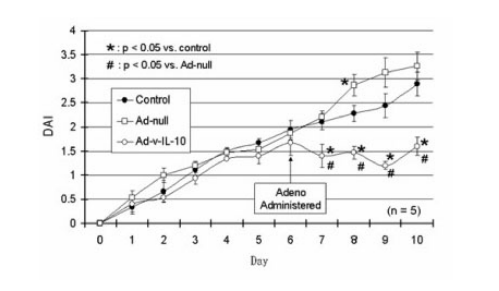
**Disease activity in mice with experimental colitis given adenoviral IL-10 gene**. Disease activity in mice in which experimental colitis was induced by feeding 3% DSS was significantly attenuated at days 7–10 when adenoviral administration of IL-10 was given on day 6. Disease activity in control mice continued at the same level as the adeno-null mice on DSS. Disease activity was slightly higher in adeno-null mice which was significant at day 8, suggesting that adenoviral infection produces some inflammation. This is important to note since Ad-IL-10 still promotes protection despite the tendency towards higher inflammation.

A combinatorial index of disease, or disease acticvity index (DAI), defined as stool blood, stool form, and weight loss [37] was used to analyze the therapeutic benefit of adenoviral gene delivery. We found that compared to adeno-null or untreated mice, adenoviral IL-10 gene transfection after induction of clinical disease reversed the course of the disease induced by DSS (Figure 2).

### C. Body weight change in adeno IL-10 gene transfected mice during colitis

**Figure 3:**
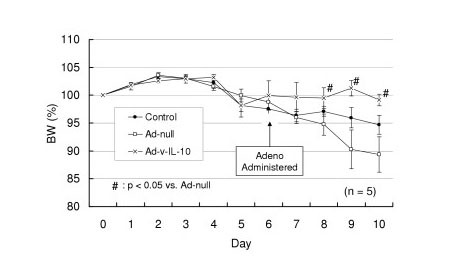
**Body weight of mice in DSS colitis**. Adeno-IL-10 mice did not lose any body weight over the course of DSS colitis, but adeno-null mice lost significantly more weight than adeno-IL-10 transfected mice (n = 5).

The anti-inflammatory effect of adenoviral IL-10 gene transfer to mice was analyzed in experimental colitis induced by feeding of oral 3% dextran sulfate (DSS, MW~40–50 kD) over the course of 10 days, and body weight recorded daily. Feeding behaviour was not altered (measured by the weight of consumed food pellets, *data not shown*). Body weight change in response to DSS was significantly different from adeno-null mice at days 8, 9 and 10 but not different from adeno IL-10 treated mice (Figure 3) consistent with a rescue from progressive weight seen in untreated mice.

### D. Colon shortening in DSS colitis and adenoviral IL-10

**Figure 4:**
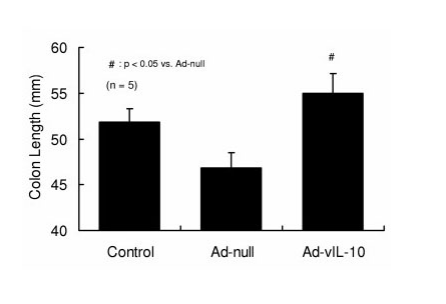
**Adeno-IL-10 blocks colon shortening induced by DSS colitis**. Adenoviral IL-10 adminstration significantly reduced the colon shortening produced by 3% DSS colitis (n = 5).

Animals fed DSS exhibited significant colon shortening compared to controls, an effect which was eliminated by adenoviral IL-10 gene transfer (Figure 4).

### E. Adenoviral IL-10 significantly lowers histopathology score in DSS colitis

**Figure 5:**
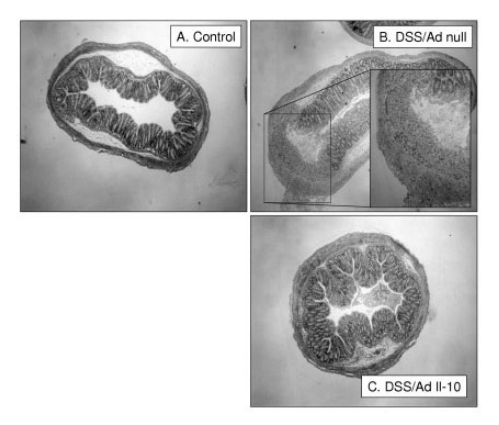
**Colon histology for adenoviral transfected mice given DSS colitis**. Figure 5A shows control colons with normal histopathology, 5B shows extensive regional leukocytic infiltration of the colon; see inset. This leukocyte infiltration is completely absent in adenoviral IL-10 treated mice which show normal or even improved morphology.

**Figure 6:**
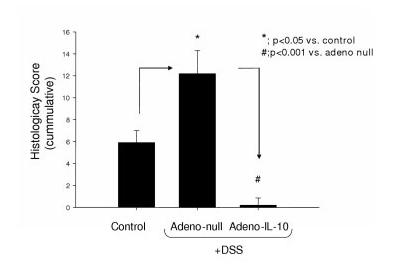
**Analysis of histopathology in adenoviral transfected DSS colitis model**. Compared to control mice, adeno-null treated mice exhibited significantly worse histopathology; whereas adeno-IL-10 treated mice had completely normal histology.

Perhaps the most remarkable finding in this study was the histopathologic effect of adeno-IL-10 on gut histopathology. Animals which had received adenoviral IL-10 vector showed virtually no evidence of any inflammation (Figure 5c), although adeno-null animals showed significant injury in response to DSS (Figure 5b) compared to controls (Figure 5a). Interestingly, the level of inflammation measured histopathologically in adenoviral IL-10 treated mice given DSS was actually lower than that measured for controls and may suggest that within the normal gut, there is a persistent, low basal level of inflammation which is normal, but that this mild inflammation can be suppressed by additional supplementation with Th2 cytokines e.g. IL-10 (Figure 6).

### F. Immunohistochemistry for MAdCAM-1

**Figure 7:**
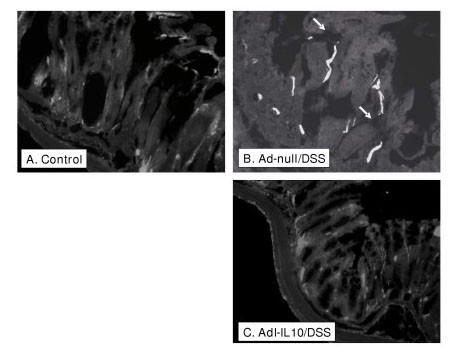
**Adenoviral IL-10 reduces MAdCAM-1 expression in experimental colitis**. Figure 7A (control) shows only sporadic and weak staining for MAdCAM-1. Figure 7B shows intense MAdCAM-1 staining in adeno-null + DSS-treated colon sections, unlike Adeno-IL10 + DSS-treated sections (Figure 7C) which lack strong MAdCAM staining, and much more closely resemble controls.

Staining of colon sections for the presence of MAdCAM-1 showed occasional staining in control treated sections. In the null adenovirus treated mice receiving DSS, colon sections showed a strong and obvious increase in MAdCAM-1 positive staining (indicated by white arrows in Figure 7b) over controls (Figure 7a), which is not observed in adeno-IL-10/DSS treated samples (Figure 7c). Image analysis revealed a large and significant increase in vessel staining from 40.33 +/- 2.79 (n = 38) in controls to 399 +/- 58.5 (n = 49); this was significantly reduced by treatment with adeno-IL-10 (79.4 +/- 22.8, n = 12) (p < 0.05, Dunnetts test).

## III. Discussion

Experimental colitis produced by DSS is thought to share many important characteristics with forms of human inflammatory bowel disease. We have previously shown that a pre-emptive induction of interleukin-10 (using a plasmid based expression vector) within endothelial cells will significantly attenuate the expression of MAdCAM-1, an important adhesive determinant which contributes to the development of human IBD, in response to TNF-a [38]. These effects may be due to enhanced endothelial barrier function [39], or to effects on adhesion molecules e.g. MAdCAM-1 and other endothelial cell adhesion molecules [4]. This is further supported by in vivo studies where animals genetically deficient in IL-10 develop spontaneous colitis with many of the characteristics of human IBD and clinical studies where IL-10 has shown some benefit in the treatment of human IBD [40].

Although many experimental therapies have been shown to be effective at preventing the induction of experimental colitis, it has of course proven more difficult to reduce an existing level of inflammatory bowel disease, since the disease process may be highly complex and difficult to control by altering a single mediator. However, models which can demonstrate effective attenuation of existing disease may provide the most relevant and important models of how human disease can be treated [41].

We showed that an adenoviral IL-10 expression vector is capable of producing very high levels of IL-10 within the peritoneal compartment, the bulk of which appears to remain confined to the peritoneal cavity, since IL-10 is not detected in plasma or serum samples following adenoviral transfection. Expression of MAdCAM-1 has also been reported in the brain, and in the heart; based on these findings, it has now been suggested that MAdCAM-1 might play roles in chronic inflammation of these organs as well [42,43].

In normal biology and especially during active inflammatory bowel disease, MAdCAM-1 may be essential to the lymphocyte homing to mucosa associated lymphoid tissue (MALT) [5,44]. Since MAdCAM-1 is normally expressed mainly within the gut microvasculature, and is dramatically amplified during IBD, it has been suggested that increased MAdCAM-1 expression contributes to the etiology of IBD through its ability to direct homing of lymphocytes to the gut. This notion is well supported by several reports that show that antibodies directed against either MAdCAM-1, or its lymphocyte ligand, the α4β7 integrin, will significantly attenuate several indices of gut damage in experimental models of colitis [8,46]. Furthermore, clinical studies conducted by Feagan et. al (2005) indicate that a humanized antibody against α4β7, an important MAdCAM-1 ligand administered to patients with active ulcerative colitis, effectively reduced the severity of the disease in comparison to those patients who received the placebo [47].

Several studies have indicated that T helper (Th1) immune responses have important roles in the development of IBD [48-50]. Moreover, dysregulation of cytokine networks is involved in Th1-dominant immune responses in IBD. Among the Th1 cytokines, TNF-α is thought to be perhaps the most important cytokine responsible for driving the onset and evolution of IBD. Because of this prime role of TNF-α in IBD, anti-TNF-α antibody therapy has been very successfully used in IBD to reduce both colonic injury and expression of ECAMs in IBD [51].

IL-10, a cytokine produced by activated macrophages and Th2-type T cells, has crucial inhibitory effects on the Th-1 type immune response, as well as on the antigen-presenting function of monocytes and macrophages [15,16]. IL-10 appears to play an important role in preventing the onset of IBD, since animals deficient in IL-10 develop colitis spontaneously, and low levels of IL-10 are positively correlated with recurrences of Crohn's disease [25,52]. However, unlike TNF-α based therapies, administration of recombinant IL-10 (rIL-10) shows poor efficacy. This may reflect the fact that TNF-α therapies for IBD are aimed at efficiently clearing TNF-α, while IL-10 therapies must *increase *IL-10 and recombinant IL-10 is likely too rapidly cleared from the circulation after *in vivo *administration to produce a uniform protection [53]. On the other hand, IL-10 *gene transfer *technology has been used with some success in models of colitis, however its efficacy is variable. One reason for this variability may be that the final serum IL-10 concentration of gene-transfected mice is below the threshold level needed for gut protection [53,54]. Therefore targeting of the IL-10 gene to the inflamed colon or its compartment should ideally exploit tissue (i.e. gut) specific promoters to control selective organ gene transfer technology, endothelial specific promoters and also organ specific intra-arterial injection of vector to activate some genes in specific locations [55].

Administration of IL-10 *in vitro *prevents TNF-α stimulated expression of MAdCAM-1 and also blocks lymphocyte adhesion on endothelial cells to the same level as dexamethasone treatment, currently a key therapy in IBD [36]. While it has been previously shown that delivery of IL-10 to the endothelium *in vitro *is protective against TNF-α [36], in vivo administration of IL-10 may be less effective [33]. Therefore methods like endothelial gene transfection in vivo may effectively maintain adequate IL-10 concentrations at the endothelial surface to finally achieve protection not obtained with intravenous IL-10 administration.

The most important index of efficacy for gene mediated recombinant IL-10 delivery in IBD is the effective inhibition of the lymphocyte-endothelium interaction mediated by MAdCAM-1. In this experiment, IL-10 induction in the endothelium efficiently blocked TNF-α induced MAdCAM-1 expression and α4β7-dependent lymphocyte adhesion on SVEC endothelial cells. Although we have not used tissue specific promoters, their use might permit even greater organ selective transgene delivery.

Our findings suggest that lymphatic or gut endothelial transfection with Th2 cytokines like IL-10 may be an effective method to reduce important symptoms associated with IBD.

## IV. Experimental procedures

### A. Adenoviral IL-10 gene transfer

#### Adenoviruses

The AdvIL-10 construct was a generous gift from Thomas Ritter, Institute of Medical Immunology, Charite-Campus Mitte, Humboldt University, Berlin, Germany. The control Ad-null construct, consisting of an E1a deleted Ad with no CMV promoter and no transgene cassette, was provided by Canji, Inc. (ZZNB; San Diego, CA). High titer adenoviral stocks were propagated in 293 cells and purified by cesium chloride gradient centrifugation. Banded virus was removed, desalted by dialysis in storage buffer (1 M sucrose, 5 mM alpha-cyclodextrin (Sigma) in PBS), and stored in small aliquots at -80°C. Repeated freeze/thaw cycles of the Ad stocks were avoided. Viral stocks and infected cells were handled only in a Class II laminar flow hood and maintained in a CO_2 _incubator designated for that purpose. The concentration of total viral particle numbers (PN) was determined by measuring the absorbance of the stocks at 260 nm. Infectious PNs were determined by measuring the concentration of viral hexon protein-positive 293 cells after a 48-h infection period. Multiplicity of infection (m.o.i.) was determined using an Adeno-X Rapid Titer Kit (Clonetics).

### B. Evaluation of Clinical Colitis

The mice were C57B6 mice, males which were obtained at 6–8 weeks of age, and used at 8–10 weeks of age, with an average weight of 23 g at the beginning of the experiments. Mice were fed either water or 3% DSS as previously described, [56]. In all animals, weight, stool blood, presence of gross blood and stool consistency were determined daily as previously described [37]. Disease activity index (DAI) was determined by combining scores of a) weight loss b) stool consistency and c) bleeding (divided by 3). Each score was determined as follows, change in weight (0:<1%, 1: 1–5%, 2: 5–10%, 4:>15%), stool blood (0: negative, 2: positive) or gross bleeding (4), and stool consistency (0: normal, 2: loose stools, 4: diarrhea) as previously described [57]. Bodyweight loss was calculated as the percent difference between the original bodyweight and the actual bodyweight on any particular day. Typically in DSS colitis animals will lose 10–15% body weight over the course of 10 days. The appearance of diarrhea is defined as mucus/fecal material adherent to anal fur. The presence or absence of diarrhea was scored as either 1 or 0, respectively, and the cumulative score for diarrhea was calculated by adding the score for each day and dividing by the number of days of exposure. Rectal bleeding was defined as diarrhea containing visible blood/mucus or gross rectal bleeding and scored as described for diarrhea. Occult blood was detected using the 'Coloscreen' (Helena Laboratories, Beaumont, TX). At the end of these studies mice were anesthetized with high dose ketamine/xylazine (200 ul/animal) with carbon dioxide asphyxia prior to collection of tissues.

### C. MadCAM-1 Immunohistochemistry

3 mm sections of tissue were frozen in OCT embedding compound and 15 um frozen sections collected onto 1% gelatin coated slides. Sections were incubated in 1:100 diluted primary anti-mouse MAdCAM-1 antibody in 0.1% milk powder in PBS for 12 h, washed 3× in this buffer, incubated in 1:1000 goat anti-rat Cy3 labeled antibody for 1 h, washed 3× and then mounted in Vectashield (Vectorlabs, Burlingame, CA). Images were analyzed for vessel staining (area) using the Image-J software package (NIH, Bethesda, MD, ).

### E. Morphological analysis

Mice were killed on day 10 of the experiment, organs were removed and fixed in 3.7% phosphate buffered formaldehyde. Sections of the distal colons were cut into 1 cm pieces and then embedded in epon/aryldite (Ted Pella). General histological assessment and scoring was carried out on sections stained using haematoxylin and eosin.

### F. Histological scoring

Histological scoring was performed on operator blinded sections using the standardized histological point system described by Cooper et al., which is used routinely for histological scoring of IBD severity [57]. A score of 0 reflects normal epithelium, without blunting, normal crypt appearance, low monocyte infiltration, and low or absent neutrophil infiltration. Three serial sections of five to six different sites of the colon (accounting for up to 18 sections per mouse) were examined at 200 × magnification; the most affected part was scored, ulceration being considered the worst lesion. A score of 1 indicates loss of single epithelial cells, mild blunting of the epithelium, single inflammatory cell infiltration of crypts, slight monocyte and neutrophil infiltrate; a score of 2 signifies loss of multiple epithelial cells (in patches), obvious flattening of the epithelia, cryptitis, and a moderate increase in monocytes and neutrophils; a score of 3 indicates frank epithelial ulceration with crypt abscesses and a marked increase in monocyte/neutrophils.

### G. Statistical analysis

All values are expressed as mean ± SD. Data were analyzed using multiple comparisons. Probability (*P*) values of <0.05 were considered significant.

## Competing interests

The author(s) declare that they have no competing interests.

## Authors' contributions

Author 1 (MS) carried out the animal studies, Author 2 (JMM) prepared the adenovirus used in these studies. Author 3 (MHJ) and 6 (TA) participated in visual sample processing and analysis. Authors 4 (PJ) and 5 (YW) helped conceive and design animal studies. Author 7 (TJ), 5 (YW) and 8 (JSA) conceived and designed the study.
